# Dataset of middle cerebral artery blood flow stability in response to high-definition transcranial electrical stimulation

**DOI:** 10.1016/j.dib.2022.108603

**Published:** 2022-09-15

**Authors:** Luiz H. Stefano, Diandra B. Favoretto, Diego C. Nascimento, Luan R.A. Santos, Marom Bikson, Joao P. Leite, Octavio M. Pontes-Neto, Dylan J. Edwards, Taiza G.S. Edwards

**Affiliations:** a Department of Neurosciences and Behavioral Sciences, Ribeirao Preto Medical School, University of Sao Paulo, Campus Universitário - CEP 14049-900, Ribeirao Preto, São Paulo, Brazil; b Departamento de Matemática, Faculdad de Ingeniería, Universidad de Atacama, Copiapó, Chile; c Neural Engineering Laboratory, Department of Biomedical Engineering, The City College of New York of the City University of New York. New York, NY, United States; d Moss Rehabilitation Research Institute, Thomas Jefferson University, PA, USA, Exercise Medicine Research Institute, School of Medical and Health Sciences, Edith Cowan University, Joondalup, Australia

**Keywords:** High-definition transcranial electric stimulation, Transcranial doppler, Hemodynamic response, Safety, HD-tDCS, high-definition transcranial direct current stimulation, TPJ, temporo-parietal junction, MCA-BFv, middle cerebral artery blood flow velocity, TCD, transcranial doppler ultrasound

## Abstract

This supplementary dataset is supportive of the randomized sham-controlled, double-blind, crossover clinical trial investigating polarity- and intensity-dependent effects of high-definition transcranial electrical stimulation (HD-tDCS) applied over the right temporo-parietal junction on mean middle cerebral artery blood flow velocity (MCA-BFv) bilaterally. Data of eleven healthy right-handed adults (6 women, 5 men; mean age 31 ± 5.6 years old) were analyzed for MCA-BFv, assessed using transcranial doppler ultrasound on the stimulated and the contralateral hemisphere concomitantly, during and after 3 blocks of 2 min HD-tDCS at 1, 2, and 3 mA. Participants received three electrical stimulation conditions (anode center, cathode center, and sham) randomly ordered across different days. The collected data is publicly available at Mendeley Data. This article and the data will inform future related investigations and safety analysis of transcranial non-invasive brain stimulation.

**Specifications Table**

**Table:** 

Subject	Neuroscience: General
Specific subject area	Transcranial Neuromodulation and Vascular Neurology.
Type of data	Table Image
How the data were acquired	The transcranial Doppler (TCD) ultrasound data was acquired using a Multidop®X; Compumedics, Germany device to assess the mean right and left middle cerebral arteries' blood flow velocity (MCA-BFv), through the transtemporal bone windows of the participants. At the same time, the HD-tDCS (Soterix Medical, New York, USA) was placed over the right temporo-parietal junction (TPJ), and participants received three electrical stimulation conditions (anode center, cathode center, and sham) on three different days. Data were recorded before (baseline), during, and after the stimulation.
Data format	Raw Analyzed Image
Description of data collection	The data of eleven healthy adults were collected in a hospital room dedicated to neurophysiological investigation, with the participant in a comfortable supine position, exposed to low light, and in silence. Bilateral MCA-BFv were concomitantly assessed using two TCD transducers fixed with a headpiece (Multidop®X; Compumedics, Germany), observed in the ultrasound display and video recorded. The time points of TCD data assessment were before, during, and after each stimulation trial. Visual analog scale data for discomfort was collected after each stimulation intensity block. The participants, assessor, and statistician were blinded to the stimulation protocol.
Data source location	Institution: Department of Neurosciences and Behavioral Sciences, Ribeirao Preto Medical School, University of Sao Paulo. City/Town/Region: Ribeirao Preto / São Paulo. Country: Brazil. Latitude: 21° 10′ 36″ South / Longitude: 47° 49′ 15″ West.
Data accessibility	https://data.mendeley.com/datasets/64n584n2d4/1 doi:10.17632/64n584n2d4.1
Related research article	Stefano LHS, Favoretto DB, Nascimento DC, Santos LRA, Louzada F, Bikson M, et al. Middle cerebral artery blood flow stability in response to high-definition transcranial electrical stimulation : A randomized sham-controlled clinical trial. Clin Neurol Neurosurg. 2022;220(May):107,345. https://doi.org/10.1016/j.clineuro.2022.107345

**Value of the Data**

• The high-definition transcranial direct current stimulation (HD-tDCS) is an emergent experimental treatment for recovering cognitive and motor deficits following neurological disorders through the modulation of cortical excitability [1,2]. As different cerebral blood flow responses can occur following neuronal activity changes, the analysis of hemodynamic effects of the main cerebral arteries is significant for applying HD-tDCS [3].
• The data were obtained using the well-established transcranial Doppler ultrasound method to assess cerebral blood flow velocity of the right and left middle cerebral arteries.
• The dataset allows anyone to reproduce the analysis published in the companion paper entitled “Middle cerebral artery blood flow stability during and after high-definition transcranial electrical stimulation: a randomized sham-controlled clinical trial”.
• The dataset can benefit researchers aiming to analyze the safety and vascular modulatory potential of non-invasive brain stimulation.

## 1 Data Description

The dataset is in a workbook (.xls) accessible in Mendeley data (see Specifications table). This dataset is composed of 14 variables described in a separate legend sheet.

**Table 1.** Raw data of right and left Middle Cerebral Artery Blood Flow velocity (MCA-BFv) and Visual Analog Scale Score for discomfort for each HD-tDCS condition and current intensity.

## 2 Experimental Design, Materials and Methods

For methodical detail of the protocol, see the companion paper “Middle cerebral artery blood flow stability during and after high-definition transcranial electrical stimulation: a randomized sham-controlled clinical trial.” [4]. In brief, we assessed the mean middle cerebral arteries' blood flow velocity (MCA-BFv) of eleven healthy right-handed adults (6 women; mean age 31 ± 5.6 years old) before, during, and after high-definition transcranial direct current stimulation (HD-tDCS). The participants, assessor, and statistician were blind to the intervention.

The HD-tDCS device (Soterix Medical, New York, USA) was controlled by an independent investigator specialized in non-invasive neuromodulation that set and delivered the intervention condition to guarantee the allocation concealment. The stimulation electrodes were placed over the right temporo-parietal junction (TPJ) region and composed of four circular Ag/AgCl electrodes (1 cm radius, 5.99e7 S/m) attached to plastic holders, filled with conductive gel (4 mm thickness, 1.4 S/m) and fixed by a HD cap. The position of the electrodes was selected after the analysis of finite element models of HD-tDCS over the targeted TPJ that predicted the induced electric field on the brain [6]. The center electrode was placed in the circumcenter of a triangle with vertices on the 10–20 electroencephalogram (EEG) system [5] coordinates C4, P4, T8, and surrounded by three electrodes placed over C4, P4, and T8. The stimulation was administered in 3 blocks of 2 min at each current intensity (1, 2, and 3 mA) and an inter-stimulus interval of 5 min between blocks. Participants received three electrical stimulation conditions (anode center, cathode center, and sham) with identical electrode positions, on three different days, with an interval of at least 24 h. In the active HD-tDCS conditions, the electric current ramped-up for the initial 30 s, maintained the current intensity for 2 min, and ramped-down for 30 s at the end of the stimulation. To ensure blinding of participant, the HD-tDCS device was positioned away from the participant's visual field and, in the sham condition, the current followed a ramp-up of 30 s to the randomized current intensity and a subsequent ramp-down phase of 30 s. The HD-tDCS conditions and current intensities were block-randomized (Table 1). The sham condition (anode or cathode center) was determined using simple balanced randomization. The same stimulation protocol was applied previously to investigate its effects on verticality perception, postural balance and EEG [6,7].

**Table 1:** Randomization method for the HD-tDCS condition and current intensity per session.

Randomization Code		Session 1	Session 2	Session 3
**A**	HD-tDCS condition Current intensity sequence (mA)	AC 1; 2; 3	CC 2; 3; 1	SH 3; 1; 2
**B**	HD-tDCS condition Current intensity sequence (mA)	AC 2; 3; 1	CC 3; 1; 2	SH 1; 2; 3
**C**	HD-tDCS condition Current intensity sequence (mA)	AC 3; 1; 2	CC 1; 2; 3	SH 2; 3; 1
**D**	HD-tDCS condition Current intensity sequence (mA)	CC 1; 2; 3	SH 2; 3; 1	AC 3; 1; 2
**E**	HD-tDCS condition Current intensity sequence (mA)	CC 2; 3; 1	SH 3; 1; 2	AC 1; 2; 3
**F**	HD-tDCS condition Current intensity sequence (mA)	CC 3; 1; 2	SH 1; 2; 3	AC 2; 3; 1
**G**	HD-tDCS condition Current intensity sequence (mA)	SH 1; 2; 3	AC 2; 3; 1	CC 3; 1; 2
**H**	HD-tDCS condition Current intensity sequence (mA)	SH 2; 3; 1	AC 3; 1; 2	CC 1; 2; 3
**I**	HD-tDCS condition Current intensity sequence (mA)	SH 3; 1; 2	AC 1; 2; 3	CC 2; 3; 1

AC, anode-center; CC, cathode-center; SH: sham; mA, milliampere.

The transcranial Doppler (TCD) ultrasound was assessed in a silent hospital room dedicated to the neurophysiological investigation. Participants were instructed not to drink caffeine or alcohol 24 h prior to each session. In a comfortable supine position with low light, participants were asked to stay still while data was collected from 2 MHz ultrasound transducers were placed over their right and the left temporal bone windows and fixed with a headpiece (Multidop®X; Compumedics, Germany;). The neurovascular neurologist (L.H.S.S.) supervised the TCD signal and adjusted the transducers as needed during the data collection and was blind to the stimulation randomization. The TCD relies on pulsed wave Doppler to produce a spectral waveform with peak systolic velocity (PSV) and end diastolic velocity (EDV) values. The mean flow velocity is the result of [PSV + (EDV * 2) / 3] and was chosen as the primary outcome measure since it is a central parameter in TCD with good reproducibility and less interindividual variability compared to systolic or diastolic peak velocities (for review [8], [9], [10]). The MCA-BFv were assessed at an average depth of 55 mm (± 3 mm) [8,9], shown in the ultrasound display and recorded in real-time video. The time-points of MCA-BFv assessment collected within each block of stimulation were: T0: baseline; T1/online: 30 s after stimulation (immediately after ramp-up); T2/online: 2 min (before ramp-down); T3/offline: immediately after the end of stimulation (after ramp-down); T4/offline: 5 min after stimulation. After each session, participants were asked to report the discomfort degree using a visual analog scale, graded from zero to 10 (Table 2), and any adverse effects related to the study protocol during the study period.

**Table 2:** Descriptive data of Visual Analog Scale Score for discomfort, with each HD-tDCS condition and current intensity.

Current Intensity	SHMean (SD)Median [IQR]	CCMean (SD)Median [IQR]	ACMean (SD)Median [IQR]
**Baseline**	0 ± 0 0 [0]	0 ± 0 0 [0]	0 ± 0 0 [0]
**1 mA**	0.72 ± 1.19 0 [0; 1]	2.36 ± 2.06 1 [1; 4.5]	0.63 ± 0.67 1 [0; 1]
**2 mA**	0.90 ± 1.22 1 [0; 1]	2.54 ± 2.58 2 [0.5; 3.5]	2.90 ± 2.54 2 [1.5; 4.5]
**3 mA**	1.45 ± 1.63 1 [0; 2.5]	3.81 ± 1.83 4 [3; 5]	3 ± 2 3 [1.5; 4]

AC: anode center; CC: cathode center; SH: sham; SD= standard deviation; IQR: interquartile range.

## Ethics Statements

This study was conducted according to the Helsinki Declaration requirements for human investigation and approved by the local ethics committee (HCRP Ethics Committee, protocol number 09,485,212.7.0000.5440). All participants provided written informed consent.

## CRediT Author Statement

**Luiz H Stefano:** Conceptualization, Methodology, Project administration, Data curation, Formal analysis, Investigation, Writing – original draft; **Diandra B. Favoretto:** Methodology, Data curation, Data curation, Investigation, Writing – original draft; **Marom Bikson:** Conceptualization, Methodology, Writing – review & editing; **Joao P. Leite:** Conceptualization, Methodology, Writing – review & editing; **Octavio M. Pontes-Neto:** Conceptualization, Methodology, Resources, Writing – review & editing; **Dylan J. Edwards:** Conceptualization, Methodology, Formal analysis, Visualization, Writing – review & editing;**Taiza G.S. Edwards:** Conceptualization, Methodology, Project administration, Data curation, Formal analysis, Resources, Supervision, Visualization, Writing – review & editing.

## Declaration of Competing Interest

The City University of New York has inventions on tES with M.B. as inventor. M.B. has equity in Soterix Medical and serves on the scientific advisory boards or has grants from Mecta, Halo Neuroscience, Boston Scientific and GlaxoSmithKline. The remaining authors declare that the research was conducted in the absence of any commercial or financial relationships that could be construed as a potential conflict of interest.

## Data Availability

Dataset of middle cerebral artery blood flow stability in response to high-definition transcranial electrical stimulation (Original data) (Mendeley Data).
